# Cognitive impairment is undetected in medical inpatients: a study of mortality and recognition amongst healthcare professionals

**DOI:** 10.1186/1471-2318-12-47

**Published:** 2012-08-24

**Authors:** Gustav Torisson, Lennart Minthon, Lars Stavenow, Elisabet Londos

**Affiliations:** 1 Department of Clinical Sciences, Clinical Memory Research Unit, Lund University, Simrisbanvägen 14, SE-205 02 Malmö, Sweden; 2 Department of Emergency Medicine, Skåne University Hospital, SE-205 02 Malmö, Sweden

**Keywords:** Cognitive impairment, Medical inpatients, Mortality

## Abstract

**Background:**

Detecting cognitive impairment in medical inpatients is important due to its association with adverse outcomes. Our aim was to study recognition of cognitive impairment and its association with mortality.

**Methods:**

200 inpatients aged over 60 years were recruited at the Department of General Internal Medicine at University Hospital MAS in Malmö, Sweden. The MMSE (Mini-Mental State Examination) and the CDT (Clock-Drawing Test) were performed and related to recognition rates by patients, staff physicians, nurses and informants. The impact of abnormal cognitive test results on mortality was studied using a multivariable Cox proportional hazards regression.

**Results:**

55 patients (28%) had no cognitive impairment while 68 patients (34%) had 1 abnormal test result (on MMSE or CDT) and 77 patients (39%) had 2 abnormal test results. Recognition by healthcare professionals was 12% in the group with 1 abnormal test and 44-64% in the group with 2 abnormal test results. In our model, cognitive impairment predicted 12-month mortality with a hazard ratio (95% CI) of 2.86 (1.28-6.39) for the group with 1 abnormal cognitive test and 3.39 (1.54-7.45) for the group with 2 abnormal test results.

**Conclusions:**

Cognitive impairment is frequent in medical inpatients and associated with increased mortality. Recognition rates of cognitive impairment need to be improved in hospitals.

## Background

Recognising cognitive impairment is of growing importance, as the worldwide prevalence of dementia is increasing [1]. In elderly medical inpatients this is particularly important, as cognitive impairment is a poor prognostic factor and an independent predictor of mortality [2-4]. Furthermore, cognitive impairment may be associated with undetected medical comorbidities, mental incapacity and risk of accidents at home after discharge [5-8]. In hospitals, patients with cognitive impairment may have communication difficulties when specifying their complaints or in the comprehension of discharge information [9].

Cognitive impairment is often undetected during an admission in general hospital settings [10-14]. In primary care, cognitive impairment is often underdiagnosed as well [15-19]. This has highlighted the need for cognitive screening to improve detection. In community-based studies, screening with subjective memory complaints (SMCs) have been tried with varying results, possibly due to large inconsistencies in definition of SMCs [20,21]. In hospital settings, delirium guidelines have recommended screening by performing cognitive tests on all elderly patients admitted to hospital [22]. Our aim was to assess the need for such a screening by performing cognitive tests in a general hospital population and compare the results with SMCs and recognition from healthcare professionals. To determine the risk accompanying cognitive impairment we also aimed to study its association with mortality.

## Methods

### Setting

The study was carried out at the wards of the Department of General Internal Medicine at the University Hospital MAS, in Malmö, Sweden. Data was collected from October 2009 through June 2010. At the time, the hospital was a 700-bed teaching hospital, providing healthcare to the community of Malmö and its surrounding areas. The hospital is now part of the larger Skåne University Hospital.

The Department of General Internal Medicine consisted of four wards with a similar general medical profile. The average length of stay was 6.4 days. Most patients (90-95%) were admitted through the hospital’s Emergency Department (ED), the rest were directly referred via their GPs.

The study included only the hospital’s general internal medicine wards; all medical departments with a higher degree of specialisation (Endocrinology, Angiology, Haematology, Nephrology, Gastroenterology, Rheumatology, Cardiology, the Department of Infectious Diseases and the Department of Pulmonary Diseases) were excluded. At the general internal medicine wards, patients tended to be older or to have multiple comorbidities to a larger extent than at the more specialised wards. The hospital’s bed manager, unaware of our study, designated the patients to their wards.

Whether at the ED or directly at the wards, a staff physician examined all patients on arrival, documenting presenting complaint, past medical history, drug history and examination findings. A nurse also assessed all patients when arriving at the wards. This included standardised estimates of the risk of falls and pressure sores, using the Downton Fall Risk Index and the Modified Norton Scale, respectively [23,24]. These procedures were done according to hospital policy.

### Patients

Eligible patients were 60 years or older, residing in the city of Malmö and not living in a nursing home. Occasionally, eligible patients were not available to enter the study, e.g. if patients were put in isolation due to norovirus infection.

Study personnel approached eligible and available patients on the first or second day of their stay to determine appropriateness for cognitive testing. For example, patients with terminal disease or severe aphasia were considered inappropriate. Patients were considered inappropriate at admission if a possibly reversible condition was present, such as severe delirium (incoherent speech, inability to focus attention) and/or abnormal laboratory values (Haemoglobin < 100 g/L, temperature > 38°C, C-Reactive Protein > 50 mg/L, abnormal electrolytes). These patients were assessed continuously and, if the condition resolved, they were subsequently included.

Eligible, available and appropriate patients were approached regarding consent. All included patients gave their written informed consent. If cognitive tests disclosed significant cognitive impairment, written consent from an informant was collected as well. This procedure was approved by the regional ethics committee at Lund University.

### Measurements

Three experienced research assistants (two certified occupational therapists and one registered nurse) carried out the measurements at the ward, in a private and calm environment, between 8 am and 4 pm.

### Interviews and comorbidity

Interviews were held with patients concerning living situation, family, education and access to home care. Presenting complaints noted in the charts on admission were recorded. Past medical history was extracted from the hospital’s charts, all conditions noted during the current or three preceding admissions were recorded. Frequent conditions were classified as absent/present. The list of current medications in the medical records was examined on admission and the cumulative number of drugs was noted. We used Charlson comorbidity index to obtain a standardised estimate of comorbidity [25].

### Cognitive tests

The MMSE, (Mini-Mental State Examination) and the CDT (Clock-Drawing Test) were employed [26,27]. The MMSE is scored from 0–30, with 0 points representing maximum cognitive impairment. As a cut-off, ≤ 23 points was used. In the CDT, the patients were asked to draw a clock on a sheet of paper and add the hands of the clock showing ”ten past eleven”. The CDT was scored from 0 to 5 according to Shulman, where 0 points denote maximum cognitive impairment and 5 points a perfect clock [27]. Any scoring uncertainties were discussed within the group until a consensus was reached. As a cut-off, ≤ 3 points were used. Previous studies have described a correlation between MMSE and CDT scores, with a mean correlation coefficient of 0.61, and that combining the tests gives higher diagnostic accuracy for neurocognitive disorders [27]. The CDT is also less affected by depression than the MMSE [28].

### QoL-AD

The Quality of Life in Alzheimer’s Disease (QoL-AD) scale was employed [29]. The QoL-AD contains 13 items (Physical health, Energy, Mood, Living situation, Memory, Family, Marriage, Friends, Self as a whole, Ability to do chores around the house, Ability to do things for fun, Money and Life as a whole). The items are rated from 1 to 4 where 1 represents poor, 2 fair, 3 good and 4 excellent. The rating can be done by patients and/or proxies.

### Subjective memory complaints (SMCs)

SMCs were determined in two ways. Firstly, a direct yes/no question was asked: ‘Do you think that your memory has gotten worse lately?’ Secondly, the memory item of the QoL-AD scale was used. This item was dichotomised, with a score of 1–2 denoting subjective memory complaints and 3–4 no impairment.

### Informants

If available at the hospital, informants rated the patients’ quality of life with the QoL-AD scale. As for the patients, the memory item was dichotomised and a score of 1 or 2 denoted cognitive impairment. This was done separately from the patients’ ratings.

### Recognition by staff physicians

The staff physicians’ admission notes from the first day were reviewed. Any notation of neurocognitive disorders (dementia, delirium or MCI – mild cognitive impairment) or current symptoms (disorientation, memory impairment, confusion, irrational behaviour etc.) was considered as recognition of cognitive impairment.

### Recognition by staff nurses

The nurses’ admission notes were examined. The Downton Fall Risk Index comprises a yes/no item entitled ”cognitive impairment” [23]. The Modified Norton Scale includes the item ”mental condition”, that is scored from 1–4 (with 1 representing ”no contact”, 2 ”cannot answer adequately”, 3 ”occasionally confused” and 4 ”fully oriented”) [24]. Scores other than ”no cognitive impairment” or ”fully oriented” on any of the scales was considered recognition of cognitive impairment.

### Intervention status

The included patients were also taking part in a prospective intervention study aiming to reduce hospital readmissions. Interventions included a pharmacist’s medication review and a changed discharge routine. For the present study, only intervention status (control or intervention) was recorded to rule out the possibility of this confounding the results.

### Statistical method

Patients were divided into three groups according to their results on cognitive tests, with 0, 1 or 2 abnormal test results, where patients with 1 abnormal result could have a low score either on the MMSE or on the CDT. All other baseline variables were compared between the three groups. In the primary comparison, ANOVA and chi-square tests were used where appropriate. In secondary analyses we did pairwise comparisons between groups with Bonferroni correction for multiple comparisons. The correlation between MMSE and CDT scores was determined using Spearman’s rho.

### Survival analysis

Bivariate Cox proportional hazards regressions were done separately for demographic variables, comorbidity variables and cognitive tests, adjusting for age and sex where applicable. The ‘number of abnormal cognitive tests’ variable was dummy-coded to compare 1 abnormal test vs 0 and 2 abnormal tests vs 0. The assumption of proportional hazards (that relative risk is not time-dependent) was tested using log-log plots and time-interaction tests, no violations of the assumption were found.

For the multivariable analysis a stepwise approach was carried out, using a backwards method with p >0.051 as the threshold for removal. Starting the stepwise model with all variables or only the ones with a bivariate p value of <0.25 resulted in the same final model. Exclusion of categorical variables with small cells (neurocognitive disorder) did not affect the final model. Testing for multiple collinearity revealed a moderate correlation between ‘Charlson comorbidity index’ and ‘number of drugs’ (Spearman’s rho, r = .46), exclusion of the latter did not affect the final model. For each step our model was controlled and fit the data adequately.

All calculations were done using the SPSS software (SPSS version 19.0, SPSS inc. Chicago Illinois). A two-sided p value of < 0.05 was considered significant.

## Results

### Patients

**Figure 1:**
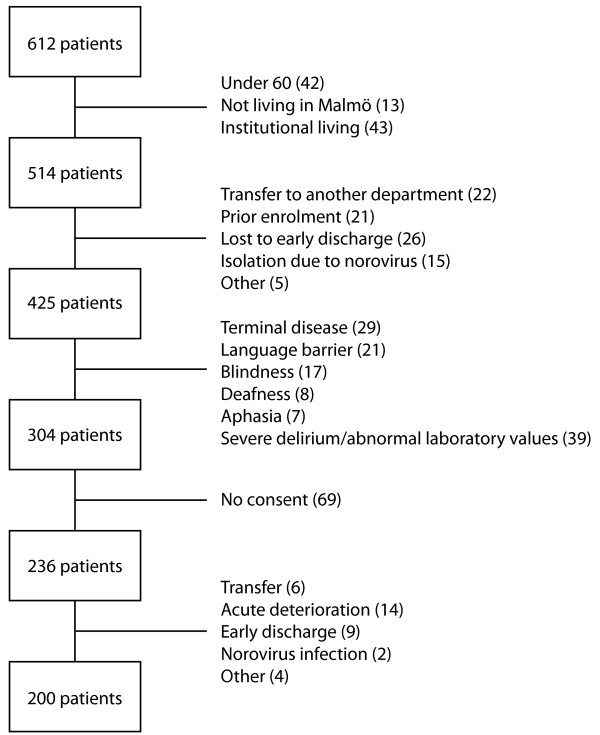
Patient flow showing exclusion criteria.

During the study period, 612 patients were admitted in total. Of these, 98 were not eligible and 89 patients were excluded because of hospital-related reasons. In 121 patients, cognitive testing was considered inappropriate. At the point of consent, 69 patients declined participation. After consent, 35 patients were excluded, due to issues occurring between consent and cognitive testing (see Figure 1). Thus, the study population consisted of 200 patients. There were no differences between the included patients, excluded patients or patients not giving consent with regard to age (ANOVA, *F* (2, 609) = 1.49, *p* = 0.23) or sex (χ^2^ (2, *N* = 612) = 3.88, *p* = 0.14).

### Results on cognitive tests

All 200 patients completed the MMSE and 198 patients completed the CDT. In total, 55 patients (28%) had 0 abnormal test results, 68 (34%) had 1 abnormal result (45 CDT and 23 MMSE) and 77 (39%) had abnormal results on both the MMSE and the CDT.

### Baseline measurements

Patients with 2 abnormal test results were older than those with 0 abnormal tests. Patients with 1 .abnormal test result were living alone to a larger extent than those with 0 abnormal test results. Regarding presenting complaints, shortness of breath was the most prevalent, accounting for nearly a third of admissions (32% in total). Falls were more frequent in the group with 2 abnormal cognitive tests than in the group with 1 abnormal test.

The mean scores on the MMSE and the CDT differed significantly between all three groups. The results on the CDT correlated moderately with the results on the MMSE (Spearman’s rho, r(198) = .50, p < .001). Baseline measurements are shown in Table 1.

**Table 1:** Baseline characteristics

	**Abnormal cognitive test results**			**Statistic**	**p value**	Significant post-hoc differences*
	**0**	**1**	**2**			
	**n = 55**	**n = 68**	**n = 77**			
Age, mean(SD)	80.6(8.8)	83.1(8.5)	85.8(6.6)	F(2, 197) = 7.45	0.001	b
Male sex	46%	30%	31%	χ^2^(2, *N* = 200) = 3.64	0.16	-
Living alone	54%	78%	68%	χ^2^(2, *N* = 200) = 6.63	0.04	a
Home care	44%	61%	62%	χ^2^(2, *N* = 200) = 4.51	0.11	-
Education ≤8 years	46%	61%	54%	χ^2^(2, *N* = 188) = 2.45	0.29	-
In intervention	50%	53%	47%	χ^2^(2, *N* = 200) = 0.56	0.76	-
Presenting complaint						
Shortness of breath	37%	33%	26%	χ^2^(2, *N* = 200) = 2.25	0.33	-
Fall	9%	3%	17%	χ^2^(2, *N* = 200) = 7.87	0.02	c
Chest pain	9%	9%	8%	χ^2^(2, *N* = 200) = 0.08	0.96	-
Infection	15%	9%	4%	χ^2^(2, *N* = 200) = 4.69	0.10	-
General condition	3%	16%	9%	χ^2^(2, *N* = 200) = 6.61	0.04	a
Pain	7%	2%	7%	FET(0vs1) *N* =123	0.17^†^	-
Neurological symptoms	2%	10%	5%	FET(0vs1) *N* =123	0.07^†^	-
Psychiatric	0%	3%	9%	FET(0vs2) *N* =132	0.04^†^	-
Laboratory value	9%	6%	5%	FET(0vs2) *N* =132	0.49^†^	-
Lower extremity symptoms	6%	7%	4%	FET(1vs2) *N* =145	0.48^†^	-
Other	2%	2%	7%	FET(1vs2) *N* =145	0.21^†^	-
MMSE, mean(SD)	26.5(2.0)	24.3(2.5)	18.9(3.2)	F(2, 197) = 143.62	(<0.001) ^††^	a, b, c
CDT, mean(SD)	4.7(0.5)	3.4(1.1)	2.4(0.9)	F(2, 195) = 109.62	(<0.001) ^††^	a, b, c

Baseline characteristics of the groups with 0, 1 and 2 abnormal cognitive tests.

* Significant differences (p < 0.05) after Bonferroni correction:

a = between 0 vs 1 abnormal cognitive test.

b = between 0 vs 2 abnormal cognitive tests.

c = between 1 vs 2 abnormal cognitive tests.

^†^ = Fischer’s exact test (FET) was used due to expected counts < 5. All three pairs (0vs1, 0vs2 and 1vs2) were compared, the comparisons with the lowest p values are displayed.

^††^ = The p values of cognitive tests are in brackets as they were the criteria for the division.

### Past medical history

Reviewing patients’ medical records showed no differences in prevalence of specific diagnoses between the three groups. Neither were there any differences in mean cumulative number of drugs in charts or in Charlson comorbidity index. In total, 14 patients had a registered ICD-10 diagnose of neurocognitive disorder (5 with Alzheimer’s disease, 4 with unspecified dementia, 4 with mild cognitive impairment and 1 with Parkinson’s disease with dementia). Of these 14 patients, 3 were found in the group with 0 abnormal test, 2 in the group with 1 abnormal test and 9 in the group with 2 abnormal tests, see Table 2.

**Table 2:** Past medical history

	**Abnormal cognitive test results**			**Statistic**	**p value**	**Significant post-hoc differences**
	**0**	**1**	**2**			
	**n = 55**	**n = 68**	**n = 77**			
Ischemic heart disease	43%	34%	27%	χ^2^(2, *N* = 200) = 3.05	0.22	-
Arrhythmia	39%	37%	30%	χ^2^(2, *N* = 200) = 1.45	0.49	-
Heart failure	24%	30%	29%	χ^2^(2, *N* = 200) = 0.81	0.67	-
Hypertension	47%	52%	47%	χ^2^(2, *N* = 200) = 0.37	0.83	-
COPD	26%	19%	16%	χ^2^(2, *N* = 200) = 2.00	0.37	-
Gastrointestinal disease	22%	18%	13%	χ^2^(2, *N* = 200) = 1.80	0.41	-
Stroke/TIA	19%	21%	21%	χ^2^(2, *N* = 200) = 0.16	0.92	-
Diabetes	22%	29%	18%	χ^2^(2, *N* = 200) = 2.63	0.27	-
Cancer, nonskin	31%	30%	25%	χ^2^(2, *N* = 200) = 0.72	0.70	-
Neurocognitive disorder	6%	4%	12%	χ^2^(2, *N* = 200) = 3.21	0.22	-
Drugs in chart, mean(SD)	7.1(3.8)	7.6(4.0)	6.6(3.7)	F(2, 197) = 1.20	0.30	-
Charlson comorbidity index, mean(SD)	2.3(1.7)	2.4(1.4)	2.1(1.5)	F(2, 197) = 0.77	0.47	-

Past medical history of the three groups. TIA = transient ischemic attack. COPD = chronic obstructive pulmonary disorder.

### Subjective memory complaints and recognition

When using the yes/no question, prevalence of SMCs was similar in all three groups, with more than two thirds of patients experiencing SMCs. When using the QoL-AD to measure SMCs, no significant differences were found between groups. Informants (n = 141) classified 46/60 (77%) of the patients with 2 abnormal tests as cognitively impaired. This was significantly more than in the other groups, in which 9/37(24%) (0 abnormal tests) and 17/44 (39%) (1 abnormal test) were classified as cognitively impaired. Staff physicians recognised 44% of the patients with 2 abnormal tests as cognitively impaired. In the same group, staff nurses recognised 64%. Neither physicians nor nurses recognised cognitive impairment in the group of patients with 1 abnormal test to a larger extent than in the group with 0 abnormal tests (Table 3).

**Table 3:** Recognition of cognitive impairment

	**Abnormal cognitive test results**			**Statistic**	**p value**	Significant post-hoc differences*
	**0**	**1**	**2**			
	**n = 55**	**n = 68**	**n = 77**			
Subjective memory complaints yes/no	67%	73%	65%	χ^2^(2, *N* = 200) = 1.45	0.48	-
Subjective memory complaints QoL-AD	48%	55%	59%	χ^2^(2, *N* = 200) = 1.74	0.42	-
Cognitive impairment recognised by informant QoL-AD	24%	39%	77%	χ^2^(2, *N* = 141) = 29.05	<0.001	b
Cognitive impairment recognised by staff physician	9%	12%	44%	χ^2^(2, *N* = 200) = 29.84	<0.001	b, c
Cognitive impairment recognised by staff nurse	15%	12%	64%	χ^2^(2, *N* = 200) = 55.44	<0.001	b, c

Recognition of cognitive impairment.

* Significant differences (p < 0.05) after Bonferroni correction:

a = between 0 vs 1 abnormal cognitive test.

b = between 0 vs 2 abnormal cognitive tests.

c = between 1 vs 2 abnormal cognitive tests.

QoL-AD = Quality of life in Alzheimer’s disease.

### Survival analysis

After 12 months, 63 patients (32%) in total were deceased with a median survival of 96 days (interquartile range 32–222). In the group with 0 abnormal tests, 8/55 (14%) were deceased, compared to 25/68 (37%) in the group with 1 abnormal test and 30/77(39%) in the group with 2 abnormal tests.

**Figure 2:**
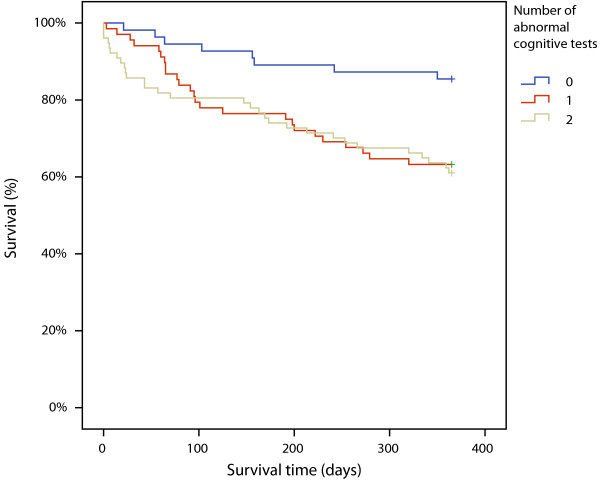
**Kaplan-Meier estimates of 12-month survival for the three groups with 0, 1 and 2 abnormal cognitive test results. **Log rank χ^2^(df = 2, *N* = 200) = 9.7, p = 0.008.

In bivariate Cox proportional hazards analysis four variables were significant predictors of mortality; home care, abnormal cognitive tests, heart failure and Charlson comorbidity index. The stepwise selection procedure resulted in a final, multivariable model in which male sex, home care, abnormal cognitive tests, and higher Charlson comorbidity scores predicted mortality. The hazard ratios (95% CI) of cognitive tests were similar for 1 and 2 abnormal tests vs 0, with 2.86 (1.28-6.39) and 3.39 (1.54-7.45), respectively, see Table 4. Kaplan-Meier estimates of survival with the number of cognitive tests as independent variable are shown in Figure 2.

**Table 4:** Cox proportional hazards

	**Bivariate models HR (95% CI)**	**p value**	**Multivariable model HR (95% CI)**	**p value**
Age (years)	1.03 (0.99-1.06)	0.11		
Male sex	1.50 (0.91-2.47)	0.11	1.75 (1.02-2.93)	0.03
Living alone	1.12 (0.63-1.98)	0.70		
Home care	1.96 (1.10-3.47)	0.02	1.82 (1.05-3.14)	0.03
Intervention status (0 = control, 1 = interv.)	1.03 (0.62-1.69)	0.92		
Education ≤ 8 years	1.03 (0.62-1.74)	0.90		
Ischemic heart disease	1.07 (0.64-1.81)	0.79		
Arrhythmia	1.21 (0.72-2.01)	0.47		
Heart failure	1.98 (1.19-3.29)	0.01		
Hypertension	0.96 (0.58-1.97)	0.87		
COPD	1.18 (0.62-2.24)	0.62		
Gastrointestinal disease	1.30 (0.71-2.40)	0.40		
Stroke/TIA	0.96 (0.51-1.82)	0.91		
Cancer, nonskin	1.44 (0.86-2.43)	0.17		
Diabetes	1.04 (0.55-2.00)	0.90		
Neurocognitive disorder	1.39 (0.60-3.23)	0.44		
Drugs (total number)	1.05 (0.98-1.11)	0.15		
Charlson index (points)	1.35 (1.16-1.58)	<0.001	1.31 (1.12-1.54)	0.001
Cognitive tests				
1 abnormal vs 0	2.98 (1.33-6.65)	0.008	2.86 (1.28-6.39)	0.01
2 abnormal vs 0	3.29 (1.47-7.45)	0.004	3.39 (1.54-7.45)	0.002

Cox proportional hazards. Bivariate models are adjusted for age and sex where applicable. All categorical variables are coded 0 = no, 1 = yes unless indicated otherwise.

## Discussion

In this study we used two cognitive tests to show that cognitive impairment was common in medical inpatients, with 73% of patients having at least one abnormal test result during hospitalisation. By using a multivariable Cox proportional hazards model, we showed that cognitive impairment was associated with a three-fold increase in 12-month mortality.

We divided the patients into three groups according to their results on cognitive tests (with 0, 1 and 2 abnormal tests). Demographic data, presenting complaints and comorbidity showed only subtle differences between the three groups. In terms of recognition, the hospital staff did not recognise cognitive impairment in the group with 1 abnormal test any more than in the group without impairment. This is alarming, as the increase in mortality risk of this group was substantial and similar to that of the group with 2 abnormal tests. To improve detection of cognitive impairment, our study suggests that subjective memory complaints are too unspecific to be used effectively, but that informants should be considered as valuable assets.

Our regression model did not retain variables that one would expect to be significant predictors of mortality, such as age or certain comorbidities, e.g. cancer. Regarding age, this could be due to the designation of patients to different departments at the hospital. At general internal medicine wards, patients tended to be either very old or slightly younger but with multiple comorbidities, for example, a 65-year old with a heart attack would typically be transferred from the ER to the cardiology department but a 65-year old with diabetes, hepatic failure, obesity, a neurologic disease and an infection would more likely be transferred to a general internal medicine ward. This selection could have affected the impact of age on mortality in our study population. Regarding comorbidities, these were coded merely as absent/present, there was no severity ranking or temporal perspective. Therefore, a patient who had had surgery 30 years ago for cancer of the colon was not distinguished from a patient with present metastatic disease. This is of course not a proper representation of the clinical situation. However, the inclusion of Charlson comorbidity index, a very stable and highly significant comorbidity variable, in our model did not affect the finding that cognitive impairment independently predicted mortality.

Studying nurses’ and physicians’ recognition at admission only may seem unfair to regular staff, as impairment could have been discovered later on. However, for ethical reasons, regular staff was informed promptly when cognitive impairment was disclosed in a patient, thus prohibiting studies of the staff’s recognition further along the admission. Changes in cognition could have occurred between admission and administration of cognitive tests, for example delirium resolving quickly or incident delirium occurring between admissions and testing. However, our cognitive tests were performed when laboratory values were within an acceptable range and patients were generally in a better state when tested than upon admission. This indicates that changes of cognition were likely to have been to the better, thus favouring the staff’s recognition on admission. The physicians and the nurses were aware of the study and it is likely that they were more vigilant towards cognitive symptoms than in regular conditions. Furthermore, we used the most generous cut-offs possible to represent recognition. Taken together, it is unlikely that the recognition rates by hospital staff have been underrated.

Representativity also needs to be addressed, as many patients were not included. Patients excluded for hospital-related reasons (with unknown cognitive status) were fewer (n = 89) than patients excluded due to disease-related conditions (n =121). The latter could be assumed more cognitively impaired, given the exclusion criteria (severe delirium, terminal disease, aphasia etc.). This would give a bias towards including the healthier part of the population. This notion is also supported by the fact that all included patients managed to fulfil the interview and the MMSE. Thus, it is unlikely that the prevalence of cognitive impairment is overrated.

We did not aim to diagnose dementia or delirium but rather to study cognitive impairment in a broader sense including its recognition and consequences in terms of mortality. To some extent, we tried to exclude patients with delirium but most likely patients fulfilling delirium criteria were included in the study. However, our findings imply that acknowledging cognitive impairment is important in medical inpatients regardless of its duration.

The main strengths of our study are its simplicity and the possibility to apply the findings in a clinical setting. We used two widely employed cognitive tests, taking approximately 15 minutes to administer. These were performed in a standardised way, much similar to that of clinical routine. We used an easily applicable approach with 0, 1 or 2 abnormal test results to make a crude estimation of cognitive impairment. Despite this simple approach, our estimate of cognitive impairment was a significant predictor of mortality in a clinical material of 200 patients with multiple diseases. Furthermore, we used simple questions and measures already applied in hospital routine to study the recognition of cognitive impairment from the patients’ perspective as well as from informants and different healthcare professionals.

## Conclusions

Our study highlights the need for improved detection of cognitive impairment in hospitals, by confirming that cognitive impairment is often underdiagnosed and associated with an increased risk of mortality. Given the high prevalence of cognitive impairment, a more active approach to identify these patients is needed. Whether mandatory cognitive tests are to be applied in all medical inpatients should be a matter of discussion and further study. It is however clear that undetected cognitive impairment is far too common in medical inpatients and recognition rates need to improve.

## Abbreviations

SMC: Subjective memory complaints; MMSE: Mini-mental state examination; CDT: Clock-drawing test; ED: Emergency department; QoL-AD: Quality of life in Alzheimer’s disease.

## Competing interests

No competing interests are declared.

## Authors’ contributions

LM and LS conceived the idea for the study. The study was designed by GT, LM, LS and EL. GT obtained data and performed statistical analysis. GT and EL drafted the manuscript, which was critically revised by LM, and LS. All authors have approved the final version of the manuscript.

## Pre-publication history

The pre-publication history for this paper can be accessed here:

http://www.biomedcentral.com/1471-2318/12/47/prepub

## References

[B1] Ferri CP Prince M Brayne C Brodaty H Fratiglioni L Ganguli M Hall K Hasegawa K Hendrie H Huang Y Global prevalence of dementia: a Delphi consensus study Lancet 2005 366 2112 2117 16360788 10.1016/S0140-6736(05)67889-0 PMC2850264

[B2] Freedberg DE Dave J Kurth T Gaziano JM Bludau JH Cognitive impairment over the age of 85: hospitalization and mortality Arch Gerontol Geriatr 2008 46 137 145 10.1016/j.archger.2007.03.006 17498822

[B3] Inouye SK Peduzzi PN Robison JT Hughes JS Horwitz RI Concato J Importance of functional measures in predicting mortality among older hospitalized patients JAMA 1998 279 1187 1193 10.1001/jama.279.15.1187 9555758

[B4] Zuccala G Pedone C Cesari M Onder G Pahor M Marzetti E Lo Monaco MR Cocchi A Carbonin P Bernabei R The effects of cognitive impairment on mortality among hospitalized patients with heart failure The Am J Med 2003 115 97 103 10.1016/s0002-9343(03)00264-x 12893394

[B5] Fu C Chute DJ Farag ES Garakian J Cummings JL Vinters HV Comorbidity in dementia: an autopsy study Arch Pathol Lab Med 2004 128 32 38 14692815 10.5858/2004-128-32-CID

[B6] Lopponen MK Isoaho RE Raiha IJ Vahlberg TJ Loikas SM Takala TI Puolijoki H Irjala KM Kivela SL Undiagnosed diseases in patients with dementia–a potential target group for intervention Dement Geriatr Cogn Disord 2004 18 321 329 10.1159/000080126 15305110

[B7] Raymont V Bingley W Buchanan A David AS Hayward P Wessely S Hotopf M Prevalence of mental incapacity in medical inpatients and associated risk factors: cross-sectional study Lancet 2004 364 1421 1427 10.1016/S0140-6736(04)17224-3 15488217

[B8] Tierney MC Charles J Naglie G Jaglal S Kiss A Fisher RH Risk factors for harm in cognitively impaired seniors who live alone: a prospective study J Am Geriatr Soc 2004 52 1435 1441 10.1111/j.0002-8614.2004.52404.x 15341543

[B9] Han JH Bryce SN Ely EW Kripalani S Morandi A Shintani A Jackson JC Storrow AB Dittus RS Schnelle J The effect of cognitive impairment on the accuracy of the presenting complaint and discharge instruction comprehension in older emergency department patients Ann Emerg Med 2011 57 662 671 e662 10.1016/j.annemergmed.2010.12.002 21272958 PMC3603343

[B10] Bowler C Boyle A Branford M Cooper SA Harper R Lindesay J Detection of psychiatric disorders in elderly medical inpatients Age Ageing 1994 23 307 311 10.1093/ageing/23.4.307 7976778

[B11] Douzenis A Michopoulos I Gournellis R Christodoulou C Kalkavoura C Michalopoulou PG Fineti K Patapis P Protopapas K Lykouras L Cognitive decline and dementia in elderly medical inpatients remain underestimated and underdiagnosed in a recently established university general hospital in Greece Arch Gerontol Geriatr 2010 50 147 150 10.1016/j.archger.2009.03.001 19359052

[B12] Harwood DM Hope T Jacoby R Cognitive impairment in medical inpatients. II: Do physicians miss cognitive impairment? Age Ageing 1997 26 37 39 9143436 10.1093/ageing/26.1.37

[B13] Inouye SK Foreman MD Mion LC Katz KH Cooney LM Nurses' recognition of delirium and its symptoms: comparison of nurse and researcher ratings Arch Intern Med 2001 161 2467 2473 10.1001/archinte.161.20.2467 11700159

[B14] Souder E O'Sullivan PS Nursing documentation versus standardized assessment of cognitive status in hospitalized medical patients Applied nursing research : ANR 2000 13 29 36 10.1016/S0897-1897(00)80016-6 10701281

[B15] Brayne C Fox C Boustani M Dementia screening in primary care: is it time? JAMA 2007 298 2409 2411 10.1001/jama.298.20.2409 18042918

[B16] Chodosh J Petitti DB Elliott M Hays RD Crooks VC Reuben DB Galen Buckwalter J Wenger N Physician recognition of cognitive impairment: evaluating the need for improvement J Am Geriatrics Soc 2004 52 1051 1059 10.1111/j.1532-5415.2004.52301.x 15209641

[B17] Olafsdottir M Skoog I Marcusson J Detection of dementia in primary care: the Linkoping study Dement Geriatr Cogn Disord 2000 11 223 229 10.1159/000017241 10867449

[B18] Lopponen M Raiha I Isoaho R Vahlberg T Kivela SL Diagnosing cognitive impairment and dementia in primary health care – a more active approach is needed Age Ageing 2003 32 606 612 10.1093/ageing/afg097 14600001

[B19] Sternberg SA Wolfson C Baumgarten M Undetected dementia in community-dwelling older people: the Canadian Study of Health and Aging J Am Geriatr Soc 2000 48 1430 1434 11083319 10.1111/j.1532-5415.2000.tb02633.x

[B20] Abdulrab K Heun R **Subjective memory impairment** a review of its definitions indicates the need for a comprehensive set of standardised and validated criteria European psychiatry : the J Association of European Psychiatrists 2008 23 321 330 10.1016/j.eurpsy.2008.02.004 18434102

[B21] Amariglio RE Townsend MK Grodstein F Sperling RA Rentz DM Specific subjective memory complaints in older persons may indicate poor cognitive function J Am Geriatr Soc 2011 59 1612 1617 10.1111/j.1532-5415.2011.03543.x 21919893 PMC3315361

[B22] Potter J George J The prevention, diagnosis and management of delirium in older people: concise guidelines Clin Med 2006 6 303 308 16826866 10.7861/clinmedicine.6-3-303 PMC4953674

[B23] Downton JH Falls in the Elderly 1993 Hodder Arnold, London

[B24] Ek AC Unosson M Bjurulf P The modified Norton scale and the nutritional state Scand J Caring Sci 1989 3 183 187 2602728 10.1111/j.1471-6712.1989.tb00290.x

[B25] Charlson M Szatrowski TP Peterson J Gold J Validation of a combined comorbidity index J Clin Epidemiol 1994 47 1245 1251 10.1016/0895-4356(94)90129-5 7722560

[B26] Folstein MF Folstein SE McHugh PR "Mini-mental state". A practical method for grading the cognitive state of patients for the clinician J Psychiatr Res 1975 12 189 198 10.1016/0022-3956(75)90026-6 1202204

[B27] Shulman KI Clock-drawing: is it the ideal cognitive screening test? Int J Geriatr Psychiatry 2000 15 548 561 10.1002/1099-1166(200006)15:6<548::AID-GPS242>3.0.CO;2-U 10861923

[B28] Manos PJ The utility of the ten-point clock test as a screen for cognitive impairment in general hospital patients Gen Hosp Psychiatry 1997 19 439 444 10.1016/S0163-8343(97)00072-8 9438188

[B29] Logsdon RG Gibbons LE McCurry SM Teri L Assessing quality of life in older adults with cognitive impairment Psychosom Med 2002 64 510 519 12021425 10.1097/00006842-200205000-00016

